# *Staphylococcus simulans* infections in a patient following high supracondylar osteotomy to treat osteoarthritis: a case report

**DOI:** 10.1093/jscr/rjae567

**Published:** 2024-09-06

**Authors:** Lin Tan, Jian Wang, Ming Li, Yadi Zhou, Binyi Xia, Minmin Zhang, Chengli Yang

**Affiliations:** Department of Pharmacy, The Affiliated Hospital of Guizhou Medical University, No. 28 Beijing Road, Yunyan District, Guiyang 550004, China; Department of Orthopedics, The Affiliated Hospital of Guizhou Medical University, No. 28 Beijing Road, Yunyan District, Guiyang 550004, China; Department of Pharmacy, The Affiliated Hospital of Guizhou Medical University, No. 28 Beijing Road, Yunyan District, Guiyang 550004, China; Department of Pharmacy, The Affiliated Hospital of Guizhou Medical University, No. 28 Beijing Road, Yunyan District, Guiyang 550004, China; Department of Pharmacy, The Affiliated Hospital of Guizhou Medical University, No. 28 Beijing Road, Yunyan District, Guiyang 550004, China; Department of Pharmacy, The Affiliated Hospital of Guizhou Medical University, No. 28 Beijing Road, Yunyan District, Guiyang 550004, China; Department of Pharmacy, The Affiliated Hospital of Guizhou Medical University, No. 28 Beijing Road, Yunyan District, Guiyang 550004, China

**Keywords:** *Staphylococcus simulans*, coagulase-negative staphylococcus, high supracondylar osteotomy, the next-generation sequencing technology

## Abstract

*Staphylococcus simulans* (*S. simulans*) is a coagulase-negative staphylococcus that is commonly found in animal pathogens. *S. simulans* rarely causes infections in clinical practice due to its low pathogenic ability and opportunistic pathogen, which results in few relevant clinical reports. In this paper, the authors primarily report a patient infected with *S. simulans* after a high supracondylar osteotomy and the *S. simulans* was identified by the means of the next-generation sequencing technology. This case report provides new evidence for the further research of *S. simulans* and paves the way for its clinical therapy.

## Introduction

*Staphylococcus simulans* (*S. simulans*) is a coagulase-negative staphylococcus (CNS), accidentally found on the skin and the urethra of healthy women [1]. Most CNS infections are caused by *Staphylococcus epidermidis*, *Staphylococcus haemolyticus*, *Staphylococci hominis*, and so on. *S. simulans* have been isolated from clinical specimens, including blood, urine, fluids and exudates those were collected from wounds, abscesses, lesions and intravascular catheters [2–4]. After a review of the literature, the authors found five cases of bone and joint infections associated with this bacterium, which were mostly reported in patients with osteomyelitis [5–9]. This case report concerned a patient with right knee osteoarthritis who developed an infection after undergoing a high supracondylar osteotomy of the right femur. The microbe was identified from infected calf puncture fluid specimens by high-throughput sequencing of the infectious pathogen.

## Case presentation

A 57-year-old female, formerly in a good health, was admitted to the affiliated hospital of Guizhou Medical University for intermittent and dull pain of the right knee longer than 2 months. The patient denied ever suffered from any system diseases such as diabetes and hypertension. Moreover, she declared no known drug allergies and no history of operation or trauma. Subsequently, the patient was diagnosed with osteoarthritis of the right knee and underwent high supracondylar osteotomy.

**Figure 1:**
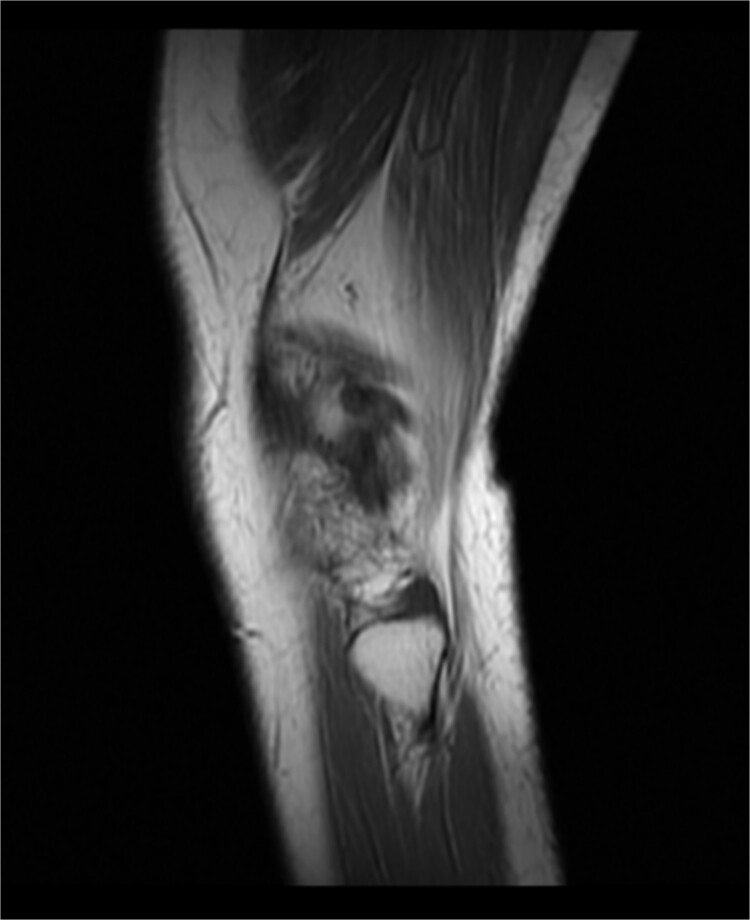
Pre-operation MRI image of the right knee joint of this patient.

Preoperative physical examination of this patient demonstrated varus deformity of right knee joint for 20° and localized swelling. Besides, the patient’s temperature was 36.5°C, while there was obvious tenderness around her right knee joint, with ROMO ° of −70°. In addition, all of the detection results including internal and lateral square stress test, drawer test and floating patellar test of this patient were negative, whereas the patella milling test was positive. The right lower extremity ends feel and muscle tone in the right lower limb of the patient was normal. Additionally, MRI scan of the right knee joint of this patient unveiled the following results: (i) the bone marrow of the patient’s right leg was swollen; (ii) the right knee medial meniscus anteriorposterior Angle II° got loss, and lateral meniscus anterioranterior Angle III° was injured; (iii) the patient suffered injuries on the anterior cruciate ligament, posterior cruciate ligament and fibular collateral ligament of the right knee; (iv) a small amount of fluid was accumulated in the right knee cavity and suprapatellar bursae (see Fig. 1). Moreover, the front and lateral chest radiographs of the patient revealed no obvious abnormalities in both lungs and thoracic vertebra degeneration. No obvious abnormality was found in both lower limb arteries and bilateral deep veins of lower limbs, and both great and small saphenous veins blood circulation was flow smoothly. Preoperative laboratory assessment results of this patient were as follows: The white blood cells (WBC) count was 6.98 × 10^9^/L (The proportion of neutrophils was 63.70%), the concentration of interleukin-6 was 7.60 pg/ml and the erythrocyte sedimentation rate was 22 mm/h.

On postoperative Day 1 (POD1), the patient’s WBC count, neutrophils percentage and C reactive protein (CRP) level soared significantly, and the patient developed a fever. In addition, clinicians and clinical pharmacists believed that the fever might be caused by the stress response. Consequently, cefuroxime was administered intravenously at a dose of 2.25 g per day to prevent infection. On the second day after surgery, the pain in the complaint area of the patient was relieved. Physical examination demonstrated that the dressing in the operative area was wrapped and fixed, no obvious abnormal exudation was observed, but the patient still suffered a fever.

**Figure 2:**
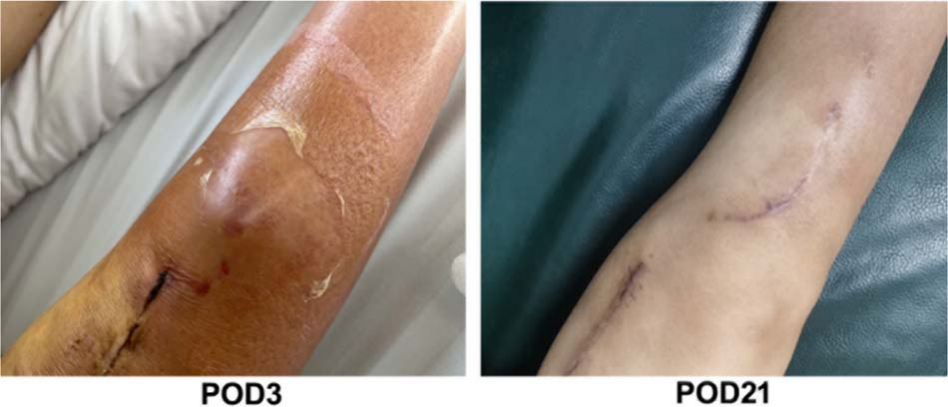
The images of physical examination result on the third and 21st day postoperation.

**Figure 3:**
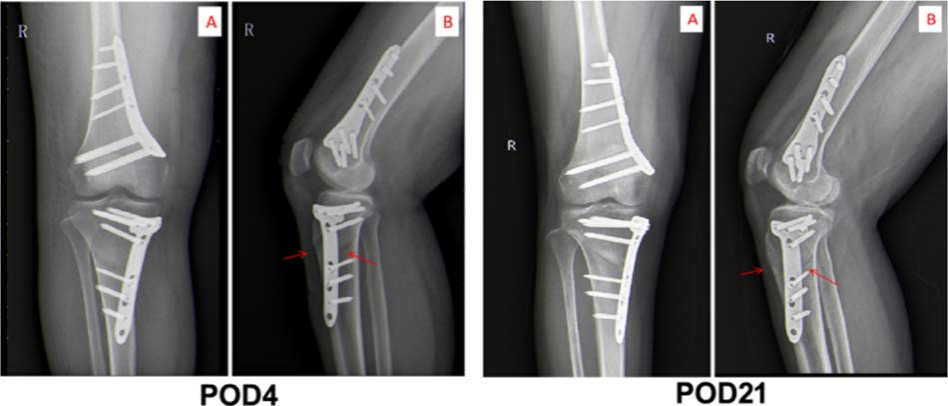
Computed axial tomography of chest. The images of CT scan on POD4 and POD21.

The third day postoperation (POD3), the patient still had a fever with a temperature up to 38.6°C. Blood test results indicated the CRP level and WBC count of this patient rose to 110.42 mg/L and 9.99 × 10^9^/L, respectively (as shown in Fig. 2). Additionally, the patient complained of incision pain and slight swelling of the lower extremities. Four days after surgery (POD4), computerized tomography (CT) scans of the right knee joint indicated degenerative changes of the joint and slight swelling of its surrounding soft tissues after osteotomy (as shown in Fig. 3). Afterwards, physical examination of the patient detected a little effusion after surgical dressing removed, and the surrounding tissue depicted a symptoms of redness, swelling and heat.

Meanwhile, considering the aggravation of infection and CT images, clinicians and clinical pharmacists stopped cefuroxime and empirically switched to vancomycin combined with meropenem. On the fifth postoperative day (the second day after the use of the dosage regimen), the patient was still feverish, with a temperature of up to 38.0°C. Consequently, the right calf puncture fluid was identified by a next-generation of high-throughput sequencing technology (Tianjin Golden Key Medical Laboratory). Sequence analysis unveiled that *S. simulans* (sequence number: 2041) was the main potential pathogen in puncture fluid. In consideration of poor anti-infection effect, vancomycin and meropenem were discontinued in this patient and replaced with linezolid to kill *S. simulans*.

After therapy, the patient’s body temperature got back to normal. In addition, the WBC count, neutrophils ratio, CRP level and interleukin-6 content were all returned to normal range. On postoperative day 13, the patient was in good condition and the excipients in the operation area were bandaged and fixed well. Additionally, the surgical incision was well closed, dry and without exudation. Furthermore, there was no redness, swelling, heat, or pain in the incision. The lower limbs were equal in length, without varus or valgus deformity, and the function of the knee flexion or extension was in good order. After discharge, linezolid was continued orally for 4 weeks until physical reexamination. Twenty-one days post-operation (POD21), the patient went back to hospital for physical re-examination. Consequently, she declared no pain in the surgical incision and no swelling of the lower extremities (Fig. 2). The CT results of reexamination indicated the following phenomenon: (i) The changes of right knee joint after osteotomy were degenerative; (ii) nodular high-density image in the lower part of the right femur, which manifests the formation of bone islands (Fig. 3).

## Discussion

Staphylococci, one of the most important bacteria that cause human diseases, can be divided into coagulase-positive and coagulase-negative according to whether secretes coagulase. Compared with coagulase-positive staphylococci, the virulence of CNS is much milder. Up to now, > 40 kinds of CNS have been identified. They usually dwell on the surface of human body but barely give rise to illness [10]. Generally, clinicians regard CNS as a contaminant of microbial culture or as part of the normal skin flora. Nevertheless, CNS is now increasingly recognized as opportunistic pathogens of severe nosocomial infections in hospital-acquired infections [11].

*S. simulans*, a kind of CNS that is noted as cause infection in animals and is rarely isolated from human skin. As a consequence, little is known about it in human pathological knowledge [12, 13]. Previous sporadic reports have manifested *S. simulans* as a cause of native valve endocarditis, vertebral osteomyelitis [13, 14]. The reason for few reports may be CNS were always considered as contaminants in the past, and clinical microbiology laboratories failed to identify it at the routine species level. Animal studies demonstrated that different from conventional CNS, *S. simulans* possesses strong pathogenicity and is easy to cause clinical infection [15, 16]. Furthermore, *S. simulans* secretes a large quantity of mucus, which allows it easier to attach on devices or foreign matters. These may explain why *S. simulans* mainly causes infection through medical equipment, implanted orthopedic devices, and blood flow [17].

In this paper, the authors firstly reported a case with *S. simulans* infection in a female patient with right knee osteoarthritis after underwent high osteotomy of supracondylar right femur. This is a particular case report, because of advances in pathogen identification and sequencing allowed us to identify the *S. simulans* as the pathogenic bacterium of this patient’s infection. The patient’s postoperative infection brought a huge burden to her postoperative rehabilitation, and reliable pathogen identification is essential for its therapy.

## Conclusion

In the past, it was generally believed that the CNS does not cause serious infections. Nevertheless, this case elaborated *S. simulans* may give rise to severe infections, and the pathogen sequencing technique is considered a gold standard for the identification of the *S. simulans*. The identification of CNS at the species level is important because accurate identification of the mimicking staphylococcus will helpful for further determine its pathogenic role in human infections. Most importantly, this case poses favorable impact on the research of severe infection caused by *S. simulans*.
